# Therapeutic Effect of the Tuber of *Alisma orientale* on Lipopolysaccharide-Induced Acute Lung Injury

**DOI:** 10.1155/2013/863892

**Published:** 2013-07-25

**Authors:** Kyun Ha Kim, Min Jung Kwun, Jun-Yong Choi, Kyung-Seop Ahn, Sei-Ryang Oh, Yong Gyu Lee, John W. Christman, Ruxana T. Sadikot, Chang Woo Han, Myungsoo Joo

**Affiliations:** ^1^School of Korean Medicine, Pusan National University, Yangsan 626-870, Republic of Korea; ^2^Korean Medicine Hospital, Pusan National University, Yangsan 626-870, Republic of Korea; ^3^Immune Modulator Research Center, Korea Research Institute of Bioscience and Biotechnology, 685-1 Yang-chung ri, Ochang, Chungbuk 363-883, Republic of Korea; ^4^Section of Pulmonary, Critical Care and Sleep Medicine, University of Illinois and the Jesse Brown Veterans Affairs Medical Center, Chicago, IL 60612, USA; ^5^Division of Allergy, Pulmonary, Critical Care and Sleep Medicine, College of Medicine, University of Florida and Malcom Randall VAMC, Gainesville, FL 32610, USA; ^6^Department of Internal Medicine, School of Korean Medicine, Pusan National University, Yangsan 626-870, Republic of Korea; ^7^Division of Applied Medicine, School of Korean Medicine, Pusan National University, Yangsan 626-870, Republic of Korea

## Abstract

Although *Alisma orientale*, an ethnic herb, has been prescribed for treating various diseases in Asian traditional medicine, experimental evidence to support its therapeutic effects is lacking. Here, we sought to determine whether *A. orientale* has a therapeutic effect on acute lung injury (ALI). Ethanol extract of the tuber of *A. orientale* (EEAO) was prepared and fingerprinted by HPLC for its constituents. Mice received an intraperitoneal (i.p.) injection of lipopolysaccharide (LPS) for the induction of ALI. At 2 h after LPS treatment, mice received an intratracheal (i.t.) spraying of various amounts of EEAO to the lung. Bioluminescence imaging of transgenic NF-**κ**B/luciferase reporter mice shows that i.t. EEAO posttreatment suppressed lung inflammation. In similar experiments with C57BL/6 mice, EEAO posttreatment significantly improved lung inflammation, as assessed by H&E staining of lung sections, counting of neutrophils in bronchoalveolar lavage fluid, and semiquantitative RT-PCR analyses of proinflammatory cytokines and Nrf2-dependent genes in the inflamed lungs. Furthermore, EEAO posttreatment enhanced the survival of mice that received a lethal dose of LPS. Together, our results provide evidence that *A. orientale* has a therapeutic effect on ALI induced by sepsis.

## 1. Introduction

Acute lung injury (ALI) is an inflammatory lung disease clinically characterized by severe hypoxemia, diffused neutrophilic infiltration to the lung, and abnormal lung compliance [1]. ALI is most often seen as part of a systemic inflammatory process, particularly systemic sepsis [2, 3]. Despite significant advances in antimicrobial therapy and supportive care to improve the survival of ALI patients, the mortality rate among ALI patients remains high, approximately 40% [4–6], and there is no effective therapeutics to treat ALI. Therefore, it is imperative to develop an effective measure against the disease.

Lipopolysaccharide (LPS), released from the outer membrane of Gram-negative bacteria, has been known as a major factor contributing to the development of ALI [7, 8]. This is because LPS causes production of proinflammatory cytokines, including tumor necrosis factor-*α* (TNF-*α*) and Interleukin-1*β* (IL-1*β*), that play a key role in progressing ALI [9]. In addition, LPS induces production of reactive oxygen species (ROS) that inflict collateral damage to tissue, exacerbating inflammation [10]. To cope with deleterious ROS, cells activate nuclear factor-E2-related factor 2 (Nrf2) [11] to express proteins that scavenge ROS, such as NAD(P)H: quinine oxidoreductase-1 (NQO-1), heme oxygenase-1 (HO-1), and glutamyl cysteine ligase catalytic units (GCLC) [12–14]. Recent studies have shown that Nrf2 also plays an essential role in protecting against acute pulmonary injury, smoke-induced emphysema, and asthma [15–17]. Thus, it is possible that Nrf2 is an excellent therapeutic target for the treatment of those inflammatory lung diseases including ALI [18, 19].

*Alisma orientale* Juzepzuk has been used as an herbal medicine for the treatment of a variety of disorders [20]. Experimental evidence suggests that *A. orientale* has anti-bacterial [21], anti-inflammatory [22–25], and antioxidative effects [26]. In addition, it was reported that *A. orientale *suppresses lung inflammation in an LPS-induced ALI mouse model [27]. However, experimental evidence to support its therapeutic effects is still scarce because those studies merely documented a preventive, but not a therapeutic, effect of *A. orientale*. Therefore, in this study, we sought to examine a therapeutic effect of *A. orientale *on ALI. For the study, we set up a new animal model, in which mice received an intraperitoneal (i.p.) injection of LPS for the induction of ALI. At 2 h after LPS injection, we administered ethanol extract of the tuber of *A. orientale* (EEAO) directly to the inflamed lungs by using microsprayer via intratracheal (i.t.) route and then determined the effect of *A. orientale* on lung inflammation incurred by ALI. We found that EEAO posttreatment reduced hallmarks in the lungs of ALI mice. In addition, EEAO posttreatment increased the survival of mice that received a lethal dose of LPS. Thus, our results show that EEAO had a therapeutic effect on ALI, suggesting that EEAO can be developed as a therapeutic, or a complementary, measure against ALI.

## 2. Materials and Methods

### 2.1. Preparation of Ethanol Extract of *Alisma orientale* (EEAO)

Dried tubers of *A. orientale* Juzepzuk were purchased from OmniHerb Corporation (Yeongcheon, Gyeonbuk, Korea), a licensed herb company, and identified by Professor K. T. Ha (School of Korean Medicine, Pusan National University, Yangsan, Republic of Korea). The voucher specimen (number: pnukh003) is kept in the herbarium stock room of the School of Korean Medicine, Pusan National University. The specimen was ground into powder, 200 g of which was added to 1,000 mL of 80% (v/v) aqueous ethanol at 60°C for 8 hours. After passing through 0.2 *μ*m filter, resultant extract was concentrated and lyophilized to yield 36 g of powder. Appropriate amounts of the powder were dissolved in phosphate buffered saline (PBS) prior to experiment.

### 2.2. Quantitative Chromatographic Analysis

To ensure the consistency of the results with EEAO, we fingerprinted the constituents of EEAO with high performance liquid chromatography (HPLC) by using Agilent 1200 Series LC System (Agilent Technologies, CA, USA) equipped with a quaternary solvent delivery pump, a vacuum degasser, an autosampler, a photodiode array detector, and Agilent ChemStation software. Chromatographic separation was conducted using an Eclipse XDB-C184.6 × 150 mm, 5 *μ*m column (Agilent Technologies, CA, USA), and isocratic elution with mobile phase of acetonitrile/water (70/30). Injection volume was 10 *μ*L with the flow rate 1.0 mL/min. Column temperature was set at 35°C. Concentrations were calculated by quantifying peak areas at the detection wavelength of 210 nm. Authentic standard of Alisol acetate B was purchased from Biopurify Phytochemical Co. Ltd. (Chengdu, Sichuan, China). Stock solution (10 mg/mL methanol) was prepared and diluted to appropriate concentration ranges to establish calibration curves. The calibration curve was linear in the range of 31.25–250 *μ*g/mL. EEAO (40 mg) dissolved in 10 mL of methanol was centrifuged at 1,000 rpm for 3 min, and the supernatant was filtered twice through 0.2 *μ*m filter before HPLC analysis.

### 2.3. Animals

Wild type C57BL/6 and transgenic mice harboring a NF-*κ*B/luciferase reporter construct were purchased from Jackson laboratory (Bar Harbor, ME, USA). All the mice were inbred in a specific pathogen-free (SPF) facility at Pusan National University, Yangsan, Republic of Korea. Animals were housed in certified, standard laboratory cages and fed with food and water *ad libitum* prior to experiment.

### 2.4. ALI Mouse Model and Survival Study

All experimental procedures followed the NIH of Korea Guidelines for the Care and Use of Laboratory Animals, and all the experiments were approved by the Institutional Animal Care and Use Committee of Pusan National University (protocol number: PNU-2010-00028). Since sepsis is the major cause of ALI [2, 3], mice received an intraperitoneal (i.p.) LPS for the induction of septic lung inflammation. Mice were anesthetized by Zoletil (Virbac, Carros cedex, France), and received a single dose of 10 mg LPS (*Escherichia coli* O55:B5 from Sigma, St. Louis, MO, USA)/kg body weight or sterile saline via an i.p. route. At 2 h after i.p. LPS administration, either PBS or EEAO (3, 30, and 300 mg/kg body weight) in 25 *μ*L of PBS was loaded in a microsprayer (Model IA-1C, Penn-Century Inc., PA, USA) and delivered in aerosol to the lung via trachea under visual guidance. At 24 h after LPS treatment, mice were euthanized by CO_2_ gas. The trachea was exposed through midline incision and cannulated with a sterile 24-gauge intravascular catheter. Bilateral bronchoalveolar lavage (BAL) was performed by two consecutive instillations of 1.0 mL of PBS. Total cell numbers in BAL fluid were counted with hemocytometer, and the cells in BAL fluid were prepared by a cytospin and stained for the differentiation of macrophages, lymphocytes, or neutrophils by Hemacolor (Merck, Darmstadt, Germany). Three hundred cells in total were counted, and one hundred of the cells in each microscopic field were scored. The mean number of cells per field was reported. For collecting lung tissue, mice were perfused with saline and the whole lung was inflated with fixatives. After paraffin embedding, 5 *μ*m sections were cut and placed on charged slides and stained with hematoxylin and eosin (H&E) staining method. Three separate H&E-stained sections were evaluated in 100x microscopic magnifications per mouse.

For survival study, mice received a lethal dose of LPS: i.p. injection of a mixture of LPS (30 mg/kg body weight) and D-(+)-galactosamine hydrochloride (500 mg/kg body weight; Sigma). At 2 h after i.p. injection of the mixture of LPS and D-(+)-galactosamine hydrochloride, mice received i.t. spraying of 30 mg/kg of EEAO. Viability of variously treated mice was monitored for up to 5 days.

### 2.5. Bioluminescence

Mice were anesthetized with Zoletil (Virbac) before imaging to immobilize them for the duration of the integration time of photon counting (3 min). Mice were shaved over the chest and abdomen before imaging. Luciferin (1 mg/mouse in 100 *μ*L isotonic saline) was administered by i.p. injection, and mice were imaged in a supine position with Optix MX3 bioimager and OptiView, an acquisition program provided by the company (ART Inc., QC, Canada). Based on our previously reported results [28], all bioluminescence measurements were done 15 min after i.p. injection of luciferin. For the duration of photon counting, mice were placed inside a light-tight box. Baseline photon counts were obtained right after LPS challenge so that each mouse could be used as its own control.

### 2.6. Total RNA Extraction and Semiquantitative RT-PCR

Total RNA was isolated from right lung homogenates with TRIZOL reagent (GeneAll, Korea) according to the manufacturer's instructions. Two micrograms of total RNA was reversetranscribed by M-MLV reverse transcriptase (Promega). Target mRNA quantity was determined by using end-point dilution PCR, including three serial 1 to 5 dilutions (1 : 1, 1 : 5, 1 : 25, and 1 : 125) of RT products for PCR amplification. The level of GAPDH (Glyceraldehyde-3-phosphate dehydrogenase) cDNA from each sample was used to normalize the samples for differences in PCR efficiency. For PCR amplification, *Taq*PCRx DNA polymerase, Recombinant (Invitrogen), and the manufacturer's protocol were used. Resultant cDNA was amplified by PCR with a set of specific primers. The forward and the reverse primers for NQO-1 were 5′-GCAGTGCTTTCCATCACCAC-3′ and 5′-TGGAGT GTGCCCAATGCTAT-3′, respectively; the primers for HO-1 were 5′-TGAAGGAGGCCAC CAAGGAGG-3′ and 5′-AGAGG TCACCCAGGTAGCGGG-3′, respectively; the primers for GCLC were 5′-CACTGCCAGAACACAGACCC-3′ and 5′-ATGGTCTGGCTGAGAAGCCT-3′, respectively; the primers for COX-2 were 5′-CCCAGAGCTCCTTTTCAACC-3′ and 5′-AATT-GGCACATTTCTTCCCC-3′, respectively; the primers for IL-1*β* were 5′-GTGTCTTTCCCGTGGACCTT-3′ and 5′-TCGTTGCTTGGTTCTCCTTG-3′, respectively; the primers for TNF-*α* were 5′-CTACTCCTCAGAGCCCCCAG-3′ and 5′-AGGCAACCTGACCACTCTCC-3′, respectively; and the primers for GAPDH were 5′-GGAGCCAAAAGGGT CATCAT-3′ and 5′-GTGATGGCATGGACTGTGGT-3′, respectively. The reaction conditions were as follows: an initial denaturation at 95°C for 5 min followed by 28 cycles of denaturation for 30 sec at 95°C, annealing for 30 sec at 58°C, and extension for 40 sec at 72°C with a final extension for 7 min at 72°C. Amplicons were separated in 1.5% agarose gels. GAPDH was used as internal controls to evaluate relative expressions of TNF-*α*, IL-1*β*, GCLC, HO-1, and NQO1. Relative expression of each gene over GAPDH was determined by densitometric analysis software ImageJ (Wayne Rasband, Research Services Branch, National Institute of Mental Health, Bethesda, MD, USA). Reactions were separated in 1.2% agarose gels in 1× TBE buffer at 100 V for 30 min, stained with SYBR safe DNA gel stain (Invitrogen), and visualized under LED light.

### 2.7. Western Blot Analysis

Nuclear proteins were isolated from the lung tissue of mice by NE-PER nuclear extraction kit and the manufacture's protocol (Thermo Scientific, IL, USA). The amounts of proteins were measured by Bradford (Bio-Rad, Hercules, CA, USA). Equal amounts of proteins were fractionated by SDS-PAGE and then transferred to PVDF membrane (Bio-Rad). Blots were blocked for at least 1 h with 5% nonfat dry milk prior to incubation with appropriate antibodies at 4°C overnight. After incubation with secondary antibodies conjugated with HRP for 1 h at room temperature, specific bands of interest were revealed by chemiluminescence (SuperSignal West Femto, Thermo Scientific).

### 2.8. Statistical Analysis

Student's *t*-test and one-way analysis of variance (ANOVA) tests with Tukey's post hoc test were applied for comparison of the means, and Kaplan-Meier estimate with log-rank test was used for survival analysis (with PASW Statistics Data Editor v18 Korean, SPSS Inc., Chicago, IL, USA), and *P* < 0.05 is considered statistically significant. All of the experiment was performed at least three times independently.

## 3. Results

### 3.1. EEAO Posttreatment Suppresses Bioluminescence in an LPS-Induced ALI Mouse Model

**Figure 1:**
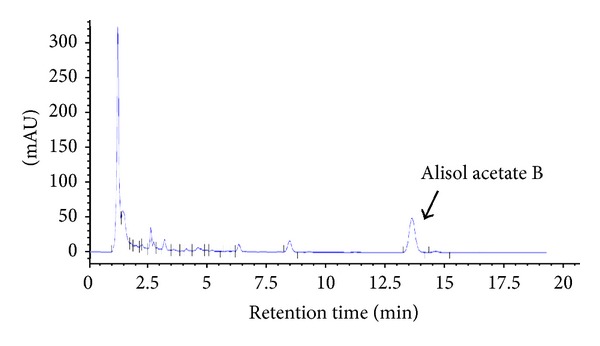
Fingerprinting of EEAO. HPLC chromatogram of EEAO was shown, where alisol acetate B, a major compound of *A. orientale,* was identified at 210 nm (RT = about 14 min). The content of alisol acetate B in EEAO was calculated on the peak-area ratio with established calibration curve.

Since previous studies have suggested the anti-inflammatory effect of EEAO [22, 24, 26] and documented a preventive effect of EEAO on neutrophilic lung inflammation in mice [27], a hallmark of ALI, we sought to examine the possibility that EEAO has a therapeutic effect on ALI. First, we prepared EEAO and fingerprinted it, along with alisol acetate B as an index component [29]. The average content of alisol acetate B in EEAO was 3.32 ± 0.01%, which was estimated on the peak-area ratio with established calibration curve (*n* = 3) (Figure 1). We used this fingerprint as a reference for the quality control of EEAO. Next, we set up a new mouse model to examine the therapeutic effect of EEAO. Most reports on the effects of herbal medicine have relied on feeding mice with herbs prior to the onset of diseases. Since prior feeding is to study preventive, rather than therapeutic, effects of traditional herbal medicine [30], we first induced septic lung inflammation via an i.p. administration of LPS (10 mg/kg) and 2 h later delivered various amounts of EEAO directly to the lung by using a microsprayer. With this devise, we could deliver EEAO in fine aerosol directly to the lung by bypassing the upper respiratory tract. We routinely delivered EEAO to 75–80% of the lung (data not shown).

**Figure 2:**
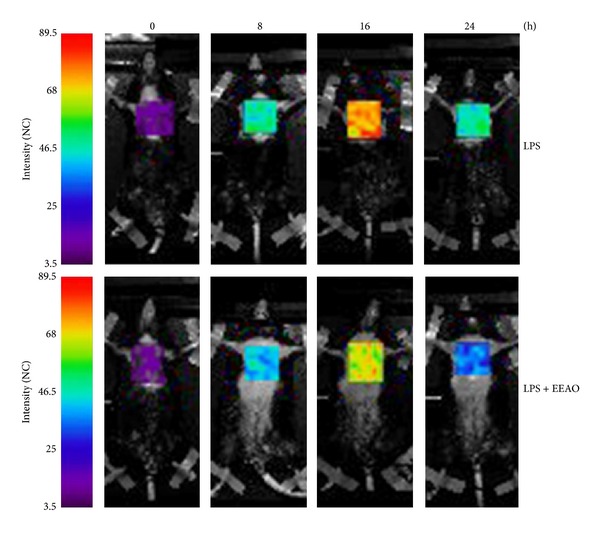
EEAO posttreatment suppresses bioluminescence in an LPS-induced ALI mouse model. Prior to i.t. spraying of EEAO (300 mg/kg body weight), NF-*κ*B reporter mice (*n* = 5 per group) received an i.p. injection of LPS (10 mg/kg body weight). At 2 h after LPS treatment, the reporter mice received an i.t. EEAO spraying to the lung. Baseline photon counts were obtained right after LPS challenge so that each mouse could be used as its own control (0 h). Bioluminescence was measured at the indicated time points after LPS injection following an i.p. luciferin injection. Shown are representative bioluminescence images of the chest of the reporter mice.

Using this system, we first determined whether EEAO posttreatment suppresses lung inflammation. Since NF-*κ*B regulates the expression of key proinflammatory cytokines [31], we used transgenic NF-*κ*B reporter mice as a surrogate for inflammatory response. Mice received an i.p. injection of LPS (10 mg/kg body weight) for the induction of ALI, and then the treated mice were divided into two groups (*n* = 5 per group). At 2 h after LPS treatment, one group received sham and the other did EEAO (300 mg/kg body weight) by i.t. spraying. This dose was based on the previously published study [27]. Bioluminescence from chest of the mice was measured right after LPS treatment (0 h), which was used as a baseline and then at 8, 16, and 24 h after i.p. LPS treatment. As shown in Figure 2, bioluminescence was progressively increased to reach a peak at 16 h after LPS administration, suggesting that an i.p. injection of LPS results in lung inflammation. In contrast, treatment of LPS-treated mice with EEAO blunted bioluminescence, suggesting that EEAO posttreatment suppresses lung inflammation.

### 3.2. EEAO Posttreatment Ameliorates Neutrophilic Lung Inflammation in LPS-Induced ALI Mice

**Figure 3:**
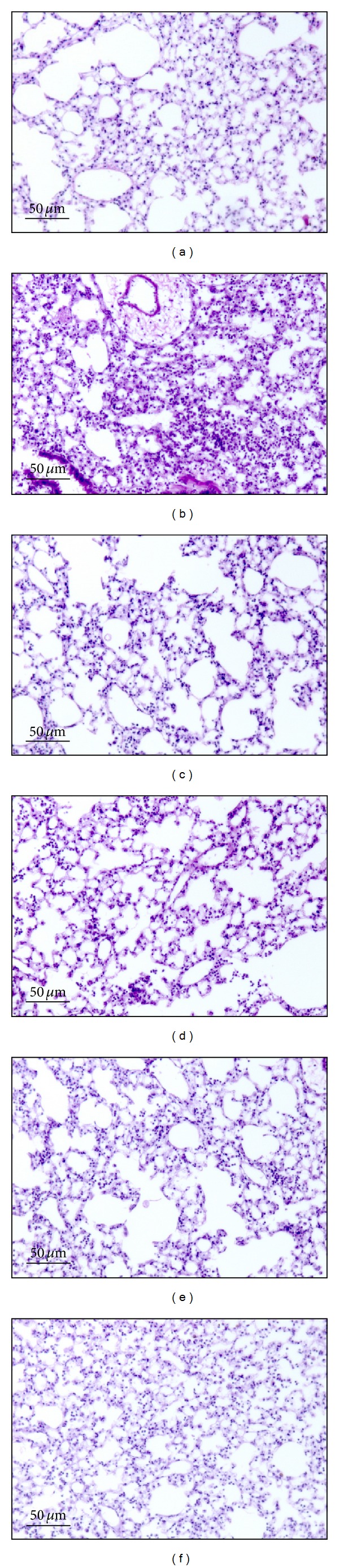
Effects of EEAO posttreatment on lung histology in ALI mice. C57BL/6 mice received an i.p. injection of PBS ((a) and (c)) or LPS ((b), (d), (e), and (f)). At 2 h after the injection, mice received an i.t. spraying of various amounts of EEAO, 300 mg/kg (c), 3 mg/kg (d), 30 mg/kg (e), and 300 mg/kg (f), or PBS ((a) and (b)) (*n* = 5 per group). At 24 h after LPS treatment, the lungs of mice were perfused, and lung sections of differentially treated mice were stained with hematoxilin and eosin (H&E) for histological examination (magnification 100x). Shown are representatives of at least five different areas of a lung.

Next, since EEAO posttreatment suppressed lung inflammation, we examined whether EEAO posttreatment has a therapeutic effect on ALI. To this end, we performed similar experiments with C57BL/6 mice (Figure 3). Mice received an i.p. injection of PBS ((a) and (c)) or LPS ((b), (d), (e), and (f)). At 2 h after the treatments, each group of mice (*n* = 5) received an i.t. spraying of PBS ((a) and (b)) or different amounts of EEAO ((c), (d), (e), and (f)). At 24 h after LPS treatment, the mice were euthanized for the analysis of lung inflammation. Lung histological analyses show that while PBS (a) or 300 mg/kg of EEAO (c) did not cause lung inflammation, LPS treatment (b) induced cellular infiltration and hyaline change in the lung. However, treatment of LPS-injected mice with increasing amounts of EEAO reduced the degrees of lung inflammation ((d), (e), and (f)).

**Figure 4:**
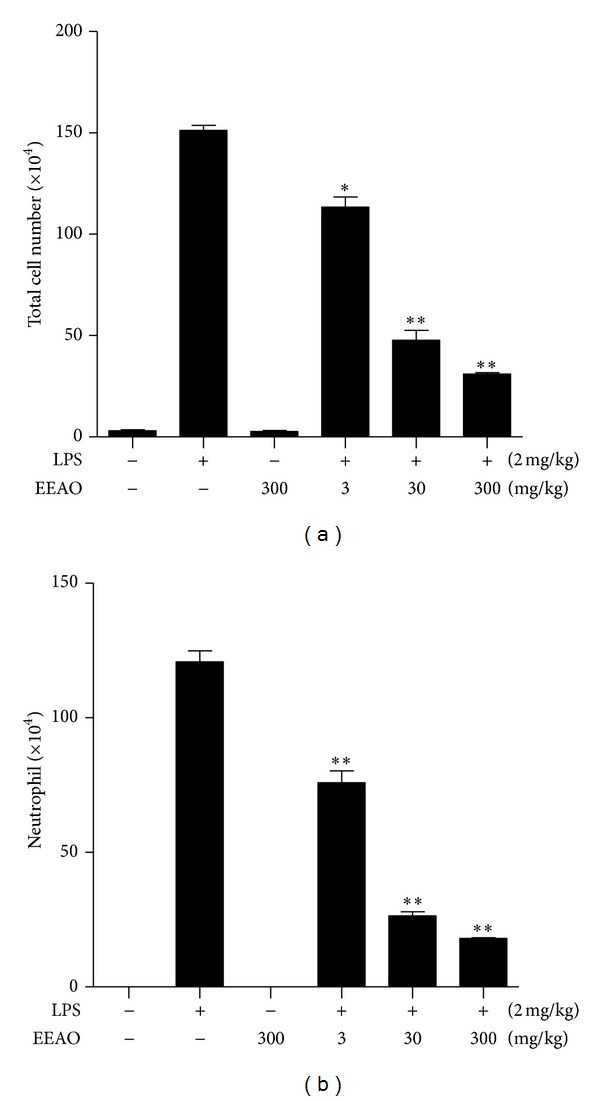
Effect of EEAO posttreatment on neutrophilic lung infiltration. Bronchoalveolar lavage (BAL) was performed to count infiltrates in the lungs of C57BL/6 mice. Mice were treated as in Figure 3. (a) From BAL fluid, total cell number was determined by using a hemocytometer. (b) The cells in BAL fluid were precipitated by a cytospin and differentially counted for neutrophils. Data represent the mean ± SEM of three independent countings. *P was less than 0.01, and **P was less than 0.05, compared with LPS treated mice (post-ANOVA comparison with Tukey's post hoc test).

To confirm the anti-inflammatory effect of EEAO, we performed bronchoalveolar lavage (BAL) of mice treated as in Figure 3, and counted cells in BAL fluid. As shown in Figure 4(a), LPS treatment increased the number of infiltrates in the lung (2nd column), which was decreased by EEAO posttreatment in a dose dependent manner (4th to 6th columns). Similarly, LPS increased the number of neutrophils infiltrated to the lung, which was decreased by EEAO posttreatment (compare 2nd with 4th to 6th columns in Figure 4(b)). It is notable that the effects by 30 mg/kg body weight of EEAO were similar to those by 300 mg/kg body weight of EEAO, suggesting that the dose of 30 mg/kg body weight of EEAO is sufficient to suppress lung inflammation.

**Figure 5:**
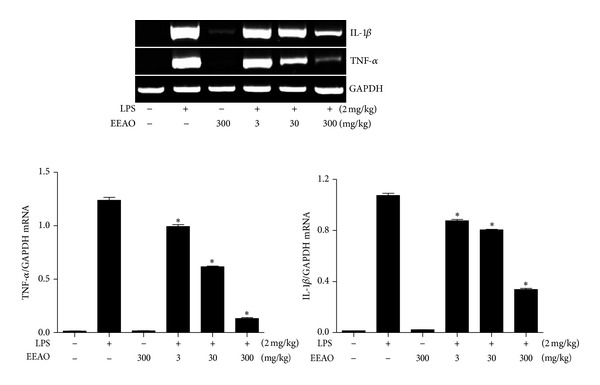
Effect of EEAO posttreatment on expressions of proinflammatory cytokines in the lung. Total RNA was extracted from the harvested lungs, and expressions of IL-1*β* and TNF-*α* were analyzed by Semiquantitative RT-PCR. The intensity of each PCR band was measured by densitometric analysis (ImageJ) and normalized to GAPDH intensity. *P was less than 0.01, compared to the LPS treated (post-ANOVA comparison with Tukey's post hoc test).

**Figure 6:**
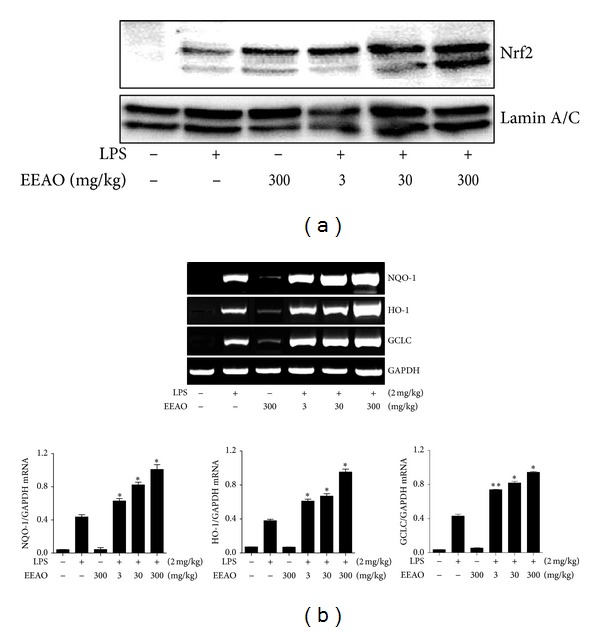
Effect of EEAO posttreatment on Nrf2 activation and Nrf2 dependent genes in the lung. (a) Nuclear proteins were extracted from the lungs of mice treated with LPS, in combination with increasing amounts of EEAO. The amounts of nuclear proteins were quantitated and analyzed by western blotting of Nrf2. The membrane was stripped and reprobed with *α*-lamin A/C for ensuring an equal loading of nuclear proteins. (b) Total RNA was extracted from the harvested lungs, and expressions of NQO-1, HO-1, and GCLC genes were analyzed by Semiquantitative RT-PCR. The intensity of each PCR band was measured by densitometric analysis (ImageJ) and normalized to GAPDH intensity. *P was less than 0.01, and **P was less than 0.05, compared with the LPS treated (post-ANOVA comparison with Tukey's post hoc test).

Since inflammation accompanies the production of proinflammatory cytokines, we examined whether the decrease of inflammatory cell infiltration is associated with decreased production of proinflammatory cytokines in the lung. The lungs of mice treated as in Figure 3 were harvested, from which total RNA was extracted for semiquantitative RT-PCR analysis of proinflammatory genes, such as IL-1*β* and TNF-*α*. As shown in Figure 5, LPS induced robust expressions of IL-1*β* and TNF-*α* (2nd lanes), which were decreased by EEAO posttreatments (lanes 4, 5, and 6). Since *A. orientale* has an antioxidant effect [26] and activates Nrf2 [27], we examined whether EEAO posttreatment enhances Nrf2 activation in the lung by western blotting of nuclear Nrf2. As shown in Figure 6(a), EEAO posttreatment enhanced Nrf2 activation. Similarly, we examined by Semiquantitative RT-PCR of the lungs whether EEAO posttreatment enhances the expression of Nrf2 dependent antioxidant genes in the lung. As shown in Figure 6(b), EEAO posttreatment enhanced the expression of Nrf2 dependent genes, such as NQO-1, HO-1, and GCLC (compare 2nd lanes with 4th, 5th, and 6th lanes). Together, these results suggest that EEAO has a therapeutic effect on lung inflammation in ALI mice.

### 3.3. EEAO Posttreatment Improves the Survival of ALI Mice

**Figure 7:**
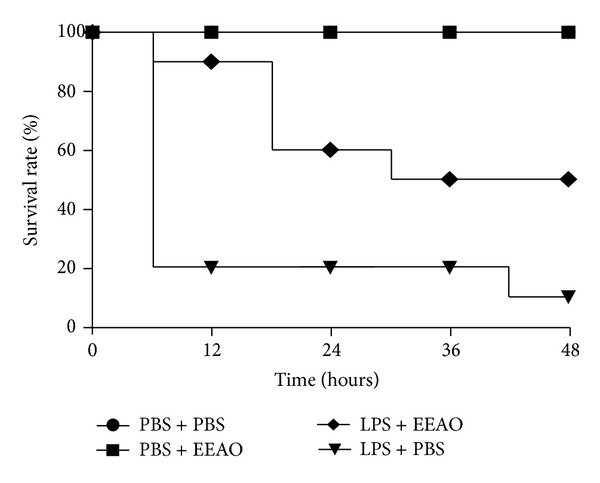
EEAO posttreatment increases the survival of septic mice. Mice received an i.p. injection of LPS (30 mg/kg body weight) and D-(+)-galactosamine hydrochloride (500 mg/kg), with or without EEAO (30 mg/kg) 2 h after the LPS treatment. Most septic mice treated with LPS and galactosamine hydrochloride (solid triangle) succumbed to death within 48 h (90%), while control mice that were treated with EEAO (solid square) or PBS (solid circle) alone all survived. On the other hand, septic mice that received EEAO 2 h after an i.p. injection of LPS and galactosamine hydrochloride (solid diamond) showed an improved survival (60% within 24 h and 50% with in 48 h after LPS injection). *N* was 20 per group, and the result is represented by Kaplan-Meier survival curves (log-rank test, *P < 0.05).

Since sepsis is the major cause of ALI [2, 3], we examined whether EEAO posttreatment protects mice from succumbing to death due to sepsis. Mice (*n* = 20 per group) were i.p. injected with a lethal dose of LPS (30 mg/kg body weight) along with D-(+)-galactosamine hydrochloride (500 mg/kg body weight) [32]. At 2 h after LPS injection, mice received an i.t. spraying of EEAO (30 mg/kg) to the lung and were monitored every 6 h for 2 days. As shown in Figure 6, the mortality of mice treated with LPS reached 80% within 12 h and 90% within 48 h. On the other hand, the mortality of septic mice that received an EEAO posttreatment was only 10% within 12 h, 40% within 24 h, and 50% within 48 h (*P* < 0.05, compared to LPS-treated mice), suggesting that EEAO offers a survival advantage to LPS-induced septic mice, see Figure 7. The mortality of all the groups remained unchanged after 2nd day up to 5th day (data not shown). Meanwhile, no mortality was observed among mice treated with PBS or EEAO see Figure 7. Together, our results suggest that EEAO has a therapeutic effect on ALI induced by sepsis.

## 4. Discussion

In this study, we examined whether the tuber of* A. orientale* has a therapeutic effect on ALI. ALI is a leading cause of death in human, with approximately 40% mortality [4–6]. Despite extensive clinical trials, there is no effective therapeutics to treat ALI, and antimicrobial therapy and supportive care to improve the survival of ALI patients are essential regimens for the patients [6, 33]. Therefore, it is imperative to develop an effective measure against the disease. Since Gram-negative bacterial infections are known as the main cause of ALI [3] and LPS is known as a key molecule that elicits inflammatory reaction [7, 34, 35], we used LPS to induce ALI in mice. To test the therapeutic effect of the tuber of* A. orientale*, we delivered ethanol extract of the tuber of* A. orientale* (EEAO) directly to the inflamed lungs of mice that received prior i.p. LPS. We found that EEAO suppressed lung inflammation incurred by ALI in mice and gave a survival advantage to the mice. Our findings provide evidence that EEAO has a therapeutic effect on ALI, suggesting that it can be developed as a therapeutic measure against ALI.

*A. orientale* is an ethnic herb that has been prescribed for a variety of diseases including oliguria, edema, gonorrhea with turbid urine, leukorrhea, diarrhea, and dizziness [20]. Recent studies show that it is also effective in renal lithiasis [36, 37], hypertension [38], hepatitis B [39], and nonalcoholic fatty liver disease [40]. In addition, anti-inflammatory [22–24, 41], antioxidative [26], and antibacterical effects of the tuber of* A. orientale* [21] have been reported. Although *A. orientale* has not frequently been prescribed for lung diseases, EEAO pretreatment was reported to suppress lung inflammation in an LPS-induced ALI mouse model. Despite experimental evidence for the various functions of* A. orientale*, these studies address mainly a preventive, but not a therapeutic, effect of the herb. Our study was designed to address a therapeutic effect of the herb on ALI. In our study, similar to the previous report, EEAO posttreatment of ALI mice was effective in suppressing neutrophilic lung infiltration, a hallmark of ALI, and mitigating inflammatory lung histology, suggesting a therapeutic effect of EEAO on ALI.

EEAO posttreatment also reduced the production of proinflammatory cytokines, TNF-*α*, and IL-1*β*. Since these cytokines contribute to neutrophilic infiltration to the lung and development of ALI [9], it is likely that the therapeutic effect of EEAO was associated with reduced production of proinflammatory cytokines. This result was consistent with previously published results demonstrating that EEAO suppresses NF-*κ*B activity and the expression of proinflammatory cytokines, such as TNF-*α* and IL-1*β*, whose expressions are governed by NF-*κ*B [27]. In addition, EEAO posttreatment enhanced the expression of Nrf2 dependent antioxidant genes, such as NQO-1, HO-1, and GCLC. Nrf2 is a transcription factor that plays a key role in protecting various inflammatory diseases including ALI [18, 42–44]. Since* A. orientale* has antioxidant effect [26], activates Nrf2, and induces the expression of Nrf2-dependent genes [27], it is also likely that enhanced expression of Nrf2-dependent genes is attributed to the therapeutic effect of EEAO. Together, these results suggest that the therapeutic effect of EEAO is associated with differential regulation of at least two key inflammatory factors, NF-*κ*B and Nrf2. 

The degrees of suppression of cellular and neutrophilic infiltration by 30 mg/kg EEAO posttreatment were similar to those by 300 mg/kg EEAO (Figure 4). In addition, the effect of 30 mg/kg EEAO on lung histology was similar to that of 300 mg/kg EEAO, and the two doses were similarly effective in maintaining normal lung histology (Figure 2). However, higher amount of EEAO was more effective in suppressing the expression of proinflammatory cytokines (Figure 5) and in enhancing the expression of Nrf2-dependent genes (Figure 6). While we did not understand this disparity, these results may indicate that strong suppression of proinflammatory cytokine production or robust induction of Nrf2-dependent gene expression is excessive, if not necessary, for curbing lung inflammation. It may suggest that 30 mg/kg EEAO was sufficient to tip the scale for the suppression of lung inflammation.

As indicated by the long history of EEAO prescribed in Asian traditional medicine, EEAO did not show any adverse effect on mice in this study. Rather, EEAO posttreatment improved the survival of mice that received a lethal dose of LPS. Given that ALI is an acute pulmonary disease accompanied by vital organ failure, it is quite intriguing that single i.t. spraying of EEAO, which is equivalent to inhalation, was effective in improving the survival of the mice. Although we did not test higher doses of EEAO for the effect on survival, it is likely that a higher dose of EEAO yielded better survival rate. Inhalation has an advantage over oral administration. It allows rapid and substantial drug absorption [30]. Thus, it can deliver efficacy with a small quantity of drugs. Given that herbal medicine administered via oral route usually takes some time to exert its effect even with high doses, our results provide evidence that inhalation of herbal medicine can be developed as an administration route for increasing the rapidity and effectiveness of herbal medicine.

## 5. Conclusion

EEAO posttreatment of mice treated with LPS was highly effective in suppressing lung inflammation. EEAO posttreatment of septic mice, which received a lethal dose of LPS, increased the survival of the mice. Thus, our results show that EEAO has a therapeutic effect on ALI induced by sepsis, suggesting the possibility that EEAO can be developed as a therapeutic means to treat acute pulmonary diseases such as ALI. In addition, our results suggest that it is feasible that inhalation of herbal medicine is an effective new administration route of herbal medicine.
